# Association between socioeconomic position and cardiovascular disease risk factors in rural north India: The Solan Surveillance Study

**DOI:** 10.1371/journal.pone.0217834

**Published:** 2019-07-08

**Authors:** Anubha Agarwal, Devraj Jindal, Vamadevan S. Ajay, Dimple Kondal, Siddhartha Mandal, Shreeparna Ghosh, Mumtaj Ali, Kavita Singh, Mark D. Huffman, Nikhil Tandon, Dorairaj Prabhakaran

**Affiliations:** 1 Department of Medicine, Northwestern University Feinberg School of Medicine, Chicago, Illinois, United States of America; 2 Centre for Chronic Disease Control, New Delhi, Delhi, India; 3 Public Health Foundation of India, Gurugram, Haryana, India; 4 Department of Preventive Medicine, Northwestern University Feinberg School of Medicine, Chicago, Illinois, United States of America; 5 The George Institute for Global Health, Sydney, Australia; 6 Department of Endocrinology and Metabolism, All India Institute of Medical Sciences, New Delhi, India; Universidad Miguel Hernandez de Elche, SPAIN

## Abstract

**Background:**

Although most Indians live in rural settings, data on cardiovascular disease risk factors in these groups are limited. We describe the association between socioeconomic position and cardiovascular disease risk factors in a large rural population in north India.

**Methods:**

We performed representative, community-based sampling from 2013 to 2014 of Solan district in Himachal Pradesh. We used education, occupation, household income, and household assets as indicators of socioeconomic position. We used tobacco use, alcohol use, low physical activity, obesity, hypertension, and diabetes as risk factors for cardiovascular disease. We performed hierarchical multivariable logistic regression, adjusting for age, sex and clustering of the health sub-centers, to evaluate the cross-sectional association of socioeconomic position indicators and cardiovascular disease risk factors.

**Results:**

Among 38,457 participants, mean (SD) age was 42.7 (15.9) years, and 57% were women. The odds of tobacco use was lowest in participants with graduate school and above education (adjusted OR 0.11, 95% CI 0.09, 0.13), household income >15,000 INR (adjusted OR 0.35, 95% CI 0.29, 0.43), and highest quartile of assets (adjusted OR 0.28, 95% CI 0.24, 0.34) compared with other groups but not occupation (skilled worker adjusted OR 0.93, 95% CI 0.74, 1.16). Alcohol use was lower among individuals in the higher quartile of income (adjusted OR 0.75, 95% CI 0.64, 0.88) and assets (adjusted OR 0.70, 95% CI 0.59, 0.82). The odds of obesity was highest in participants with graduate school and above education (adjusted OR 2.33, 95% CI 1.85, 2.94), household income > 15,000 Indian rupees (adjusted OR 1.89, 95% CI 1.63, 2.19), and highest quartile of household assets (adjusted OR 2.87, 95% CI 2.39, 3.45). The odds of prevalent hypertension and diabetes were also generally higher among individuals with higher socioeconomic position.

**Conclusions:**

Individuals with lower socioeconomic position in Himachal Pradesh were more likely to have abnormal behavioral risk factors, and individuals with higher socioeconomic position were more likely to have abnormal clinical risk factors.

## Introduction

Cardiovascular disease is the leading cause of mortality in India.[1,2] United Nations Member States agreed on selected risk factor targets to reduce premature mortality from cardiovascular and other non-communicable diseases by 25% by 2025 and by one-third by 2030.[3–5] These risk factors for cardiovascular disease such as high systolic blood pressure, high fasting plasma glucose, high total cholesterol and high body mass index (BMI) contributed about twice as many disability adjusted life years in India in 2016 compared to 1990 according to the Global Burden of Disease Study.[6] A 2017 meta-analysis of 1.7 million individuals demonstrated an inverse association between socioeconomic position and premature mortality, highlighting social determinants as a key target for improving population health.[7] To achieve progressive global health targets, a better understanding of the sociodemographic patterning of cardiovascular disease risk factors is needed in rural India, where the majority of India’s population resides.[4] To address this gap, we sought to describe the distribution and association between indicators of socioeconomic position and cardiovascular disease risk factors in a large, representative rural population in Himachal Pradesh, India.

## Methods

### Study population

We performed representative, community-based sampling from 2013 to 2014 of Solan district in Himachal Pradesh in northern India.[8] Solan district covers an area of 1,936 square kilometers consisting of mountainous terrain. Most of the district’s population (N = 580,320 based on the 2011 Census of India) lives in rural, agrarian communities working as land cultivators or agricultural laborers.[9] In collaboration with the Government of Himachal Pradesh, 38 health sub-center areas surrounding 5 government health care facilities (4 community health centers, 1 regional hospital) were selected based on their proximity to the district health care facilities. Through house-to-house sampling, all non-pregnant, consenting residents of the health sub-center areas aged 20 years or older were enrolled in the study. Demographic, social, and medical history data were collected through household interviews in Hindi by trained field research teams using a standardized questionnaire entered on an electronic tablet. Standardized clinical examinations and point-of-care fasting capillary blood glucose finger stick sampling were performed in participants’ homes. A subset of participants was selected by convenience technique to undergo venous blood samples for analysis of fasting lipid panel. Venous blood samples were analyzed in an accredited laboratory (Solan, India) that underwent external quality assessment.

The study received approval from ethics committees of Centre for Chronic Disease Control (New Delhi, India) and All India Institute of Medical Sciences (New Delhi, India). All participants provided written informed consent prior to enrollment. The Indian Council of Medical Research, Medtronic Foundation, and National Heart, Lung, and Blood Institute funded the study and were not part of the design, analysis nor decision to publish.

### Socioeconomic position

We collected self-reported data on participant education, participant occupation, household income, and household assets as indicators of socioeconomic position based on prior literature.[10–15] The highest level of education obtained was used to characterize participants into four categories: primary school and below (up to class IV, literate with no formal education, or illiterate), high school (class V to IX), secondary school (class X to XII), and graduate and above (bachelor of arts, bachelor of science, bachelor of commerce, diploma, or professional degree). Occupation was categorized as not working (i.e. unemployed, retired, or student), homemaker (i.e. a person who manages the home), low skilled (i.e. manual laborer, rickshaw driver, carpenter, etc.), and skilled (i.e. farmer, business owner, teacher, etc.). Household monthly income was stratified into four quartiles of ≤5,000 Indian Rupees (INR), 5001–10,000 INR, 10,001–15,000 INR, and >15,000 INR. The number of participants reporting monthly household income of exactly 5,000 INR or 10,000 INR was large, leading to unequal distribution of participants across quartiles. We used principal components analysis to construct a wealth index of household assets with four ordered levels (low, medium, high, highest), which incorporated different household features (separate cooking room, type of fuel used, toilet facilities, water source) and assets (television, refrigerator, washing machine, microwave, mixer-grinder, DVD player, computer, car, motorcycle and bicycle).[10,16] The components in the household asset score had a Kaiser-Meyer Olkin (KMO) statistic of 0.71. KMO values greater than 0.6 indicate that variables have enough in common to be utilized in a principal components analysis.[15]

### Cardiovascular disease risk factors

We collected data on tobacco use, alcohol use, physical activity, BMI, blood pressure, and blood glucose as cardiovascular disease risk factors. Self-reported current tobacco use included cigarette, beedi, cigar, tobacco chewing, or pan masala use in the past 6 months. Self-reported alcohol use included local spirits, beer and wine use in the past 6 months. Participants were categorized into low, medium, or high physical activity levels based on self-reported levels captured using the International Physical Activity Questionnaire.[17] BMI was calculated (weight/height squared) based on measured weight (Omron weighing scale HN-286; Omron Corporation, Kyoto, Japan) and height (seca 201 measuring tape, seca, Hamburg, Germany). We used international guidelines to define overweight (BMI 25.0–29.9 kg/m^2^) and obesity (BMI ≥ 30.0 kg/m^2^) status. Blood pressure was measured in participants’ homes by trained research staff using an automated measurement system (Omron HEM-7080 and HEM-7080IT-E; Omron Corporation, Kyoto, Japan) after the participant had been seated at rest for five minutes with participants’ feet, back, and arm supported. Two discrete blood pressure measurements were obtained, and a third measurement was obtained if there was a difference of 10 mmHg in the systolic blood pressure measures or 5 mmHg in the diastolic blood pressure measures. The mean of the blood pressure measurements (first and second measurement, or second and third measurement if assessed) was used in the analysis. Hypertension was defined as measured blood pressure ≥140/90 mmHg, on blood pressure lowering medication, or self-report during the household questionnaire assessment. We performed a sensitivity analysis defining hypertension based on measured blood pressure ≥130/80 mmHg based on thresholds derived from a recent clinical practice guideline update, use of blood pressure lowering medication, or self-report during the household questionnaire assessment (S1 Table).[18] Diabetes was defined as fasting capillary blood glucose ≥126 mg/dL, on hypoglycemic medications, or self-report during the household questionnaire assessment.

### Statistical analysis

We performed statistical analyses using Stata version 14 (StataCorp, College Station, TX, USA) and created figures using R software (version 3.3.2; R Foundation, Vienna, Austria). Missingness of participant data was low (3.9%), and we performed a complete case analysis as a result. We summarize sociodemographic characteristics and present categorical variables as frequencies with proportion (%) and continuous variables as means with standard deviation (SD). There was no collinearity between the exposure variables of participant education, participant occupation, household income, and household assets, which we evaluated separately as others have done.[19] We performed hierarchical multivariable logistic regression, adjusting for age, sex, and clustering of the health sub-centers to account for potential clustering of exposures and outcomes at this level, to evaluate the cross-sectional association of each socioeconomic position indicator and each discrete cardiovascular disease risk factor. We also performed multivariable logistic regression to evaluate the cross-sectional association of each socioeconomic position indicator and number of abnormal cardiovascular disease risk factors. We used linear regression to evaluate the cross-sectional association of each socioeconomic position indicator and systolic blood pressure, fasting plasma glucose, BMI, total cholesterol and HDL cholesterol. We present the unadjusted model, model adjusted for age and sex, and model adjusted for age, sex, and clustering of the health sub-centers to account for potential clustering of exposures and outcomes at this level. A two-sided p value <0.05 defined statistical significance.

## Results

We enrolled 40,017 participants. We excluded 1,560 participants (3.9%) with missing data in the exposures or outcomes to arrive at a complete case analysis of 38,457 participants. The characteristics of participants with missing data are presented in S2 Table. A greater proportion of excluded participants with missing data had primary school and below education (24.7% vs 20.3%, P <0.001), household income less than or equal to 5,000 INR (34.8% vs 29.9%, P <0.001), and low household assets (33.8% vs 24.7%, P <0.001). There were no differences between excluded and included participants in current tobacco use (12.2% vs 11.0%, P = 0.12) and current alcohol use (8.3% vs 7.5%, P = 0.24).

In the complete case analysis, 57.0% were women, and the mean (SD) age of participants was 42.7 (15.9) years (Table 1). For education, 44.3% of men and 32.4% of women had completed schooling up to secondary school. Mean (SD) years of formal education were 9.5 (4.5) years and 7.4 (5.2) years among men and women, respectively. For occupation, most men were not working (51.3%), and 34.5% reported having skilled jobs. Most women (85.6%) were homemakers, and 3.8% reported having skilled jobs. For monthly household income, 29.9% reported earning ≤5,000 INR and 35.3% reported earning between 5,001 and 10,000 INR. More men than women reported using tobacco (23.2% vs. 1.8%, P <0.001) and alcohol (17.3% vs <1%, P <0.001). More women than men were overweight (19.1% vs 15.1%, P <0.001) and obese (5.8% vs 2.9%, P <0.001). One out of every five participants (21.2%) had hypertension, and 4.4% had diabetes.

**Table 1:** Demographic, social, and medical characteristics of Solan Surveillance Study participants.

Characteristic	Total	Male	Female	P value
No. of participants	38,457	16,528	21,929	
Age group, n (%)				
20–29 years	9327 (24.3%)	3769 (22.8%)	5558 (25.3%)	<0.001
30–39 years	9367 (24.4%)	3885 (23.5%)	5482 (25.0%)	
40–49 years	7574 (19.7%)	3275 (19.8%)	4299 (19.6%)	
50–59 years	5549 (14.4%)	2464 (14.9%)	3085 (14.1%)	
60–69 years	3827 (10.0%)	1748 (10.6%)	2079 (9.5%)	
≥70 years	2813 (7.3%)	1387 (8.4%)	1426 (6.5%)	
Mean age, years (SD)	42.7 (15.9)	43.6 (16.3)	41.9 (15.5)	<0.001
Education^a^, n (%)				
Primary school and below	7815 (20.3%)	1891 (11.4%)	5924 (27.0%)	<0.001
High school	10,581 (27.5%)	4305 (26.0%)	6276 (28.6%)	
Secondary school	14,428 (37.5%)	7314 (44.3%)	7114 (32.4%)	
Graduate & above	5633 (14.6%)	3018 (18.3%)	2615 (11.9%)	
Mean years of formal education (SD)	8.3 (5.0)	9.5 (4.5)	7.4 (5.2)	<0.001
Occupation^b^, n (%)				
Homemaker	18,913 (49.2%)	152 (0.9%)	18,761 (85.6%)	<0.001
Not working	10,481 (27.3%)	8486 (51.3%)	1995 (9.1%)	
Low skilled	2516 (6.5%)	2185 (13.2%)	331 (1.5%)	
Skilled	6547 (17.0%)	5705 (34.5%)	842 (3.8%)	
Monthly household income^c^, n (%)				
≤INR 5,000	11,485 (29.9%)	4830 (29.2%)	6655 (30.3%)	0.06
INR 5,001–10,000	13,594 (35.3%)	5947 (36.0%)	7647 (34.9%)	
INR 10,001–15,000	5388 (14.0%)	2328 (14.1%)	3060 (14.0%)	
>INR 15,000	7990 (20.8%)	3423 (20.7%)	4567 (20.8%)	
Mean monthly household income, INR (SD)	12,355.9 (15404.0)	12,307.7 (13448.4)	12,392.3 (16727.7)	0.59
Household asset quartile, n (%)				
Low	9639 (25.1%)	4178 (25.3%)	5461 (24.9%)	0.53
Medium	10,592 (27.5%)	4493 (27.2%)	6099 (27.8%)	
High	9018 (23.4%)	3872 (23.4%)	5146 (23.5%)	
Highest	9208 (23.9%)	3985 (24.1%)	5223 (23.8%)	
Current tobacco use, n (%)	4220 (11.0%)	3832 (23.2%)	388 (1.8%)	<0.001
Current alcohol use, n (%)	2871 (7.5%)	2862 (17.3%)	9 (<1%)	<0.001
Physical activity, n (%)				
Low	1888 (4.9%)	940 (5.7%)	948 (4.3%)	<0.001
Moderate	3433 (8.9%)	1697 (10.3%)	1736 (7.9%)	
High	33,136 (86.2%)	13,891 (84.0%)	19,245 (87.8%)	
Overweight^d^, n (%)	6690 (17.4%)	2502 (15.1%)	4188 (19.1%)	<0.001
Obesity^e^, n (%)	1750 (4.6%)	482 (2.9%)	1268 (5.8%)	<0.001
Mean BMI, kg/m^2^ (SD)	22.3 (4.1)	22.1 (3.6)	22.5 (4.4)	<0.001
Mean waist circumference, cm (SD)	82.4 (11.3)	82.8 (10.3)	82.0 (12.0)	<0.001
Mean systolic blood pressure, mmHg (SD)	124.3 (16.9)	127.3 (16.0)	122.0 (17.2)	<0.001
Mean diastolic blood pressure, mmHg (SD)	79.0 (9.7)	80.2 (9.7)	78.0 (9.7)	<0.001
Mean fasting plasma glucose, mg/dL (SD)	92.6 (23.0)	92.1 (22.5)	92.9 (23.5)	<0.001
Mean total cholesterol^f^, mg/dL (SD)	183.4 (41.3)	182.3 (40.3)	184.2 (41.9)	0.05
Mean HDL cholesterol^g^, mg/dL (SD)	44.2 (10.4)	41.7 (10.6)	45.8 (9.9)	<0.001
Hypertension^h^, n (%)	8156 (21.2%)	3882 (23.5%)	4274 (19.5%)	<0.001
Diabetes^j^, n (%)	1690 (4.4%)	729 (4.4%)	961 (4.4%)	0.89

**SD**: standard deviation, **INR**: Indian rupee, **BMI**: body-mass index, **HDL**: high-density lipoprotein

^a^ Primary school and below: up to class IV, literate with no formal education, or illiterate; High school: class V to IX; Secondary school: class X to XII; Graduate & above: bachelor of arts, bachelor of science, bachelor of commerce, diploma, or professional degree.

^b^ Homemaker: a person who manages the home; Not working: unemployed, retired, or student; Low skilled: manual laborer, rickshaw driver, carpenter, etc.; Skilled: farmer, business owner, teacher, etc.

^c^ Monthly household income based on quartiles.

^d^ Overweight: BMI 25.0–29.9 kg/m^2^.

^e^ Obesity: BMI ≥ 30.0 kg/m^2^.

^f^ Measured in subset of 7752 participants.

^g^ Measured in subset of 7751 participants.

^h^ Defined as measured blood pressure ≥140/90 mmHg, on blood pressure lowering medication or by self-report.

^i^ Defined as fasting capillary blood glucose ≥126 mg/dL, on hypoglycemic medications or by self-report.

The distribution of cardiovascular risk factors by education of participants is shown in Table 2. Substance use follows an inverse, graded pattern with tobacco and alcohol use higher in men with lower education. After age-, sex-, health sub-center-adjustment, the odds of tobacco (OR = 0.11, 95% CI 0.09, 0.13) and alcohol use (OR = 0.42, 95% CI 0.32, 0.55) were lower in participants with graduate school and above education compared to primary school and below (S3 Table). Among women with lower educational attainment, mean systolic blood pressure and mean total cholesterol levels were higher. There was an inverse, graded pattern between lower educational attainment and higher, unadjusted prevalence of hypertension (30.2% men and 33.8% women with primary school and below schooling versus 21.3% men and 8.7% women with graduate school and above, P <0.001). However, after adjusting for age, sex, and health sub-center clustering, the odds of hypertension was highest in participants with graduate school and above compared with primary school and below education (OR 1.39, 95% CI 1.19, 1.62, S3 Table). A similar, inverse graded pattern was observed for women with lower educational attainment and higher, unadjusted prevalence of diabetes. After adjusting for age, sex, and health sub-center clustering, the odds of diabetes was highest in participants with graduate school and above (OR 2.15; 95% CI 1.59, 2.90; S3 Table). Although no consistent patterns were observed with the distribution of cardiovascular disease risk factors by occupation of participants, low skilled men had higher prevalence of current tobacco (27.4% versus 24.3% in men not working; adjusted OR 1.60, 95% CI 1.20, 2.15) and alcohol (21.0% versus 16.4% in men not working; adjusted OR 1.70, 95% CI 1.29, 2.23) use compared to those not working (Table 3, S4 Table).

**Table 2:** Distribution of cardiovascular risk factors by education of Solan Surveillance Study participants.

	Primary school and below		High school		Secondary school		Graduate school and above		P value^a^
	Male n = 1891	Female n = 5924	Male n = 4305	Female n = 6276	Male n = 7314	Female n = 7114	Male n = 3018	Female n = 2615	
Current tobacco use, n (%)	872 (46.1%)	362 (6.1%)	1491 (34.6%)	25 (0.4%)	1218 (16.7%)	1 (<1%)	251 (8.3%)	0 (0.0%)	P<0.001
Current alcohol use, n (%)	507 (26.8%)	7 (0.1%)	1021 (23.7%)	2 (<1%)	1033 (14.1%)	0 (0.0%)	301 (10.0%)	0 (0.0%)	P<0.001
Physical activity, n (%)									P<0.001
Low	171 (9.0%)	333 (5.6%)	204 (4.7%)	247 (3.9%)	339 (4.6%)	267 (3.8%)	226 (7.5%)	101 (3.9%)	
Moderate	256 (13.5%)	621 (10.5%)	408 (9.5%)	451 (7.2%)	677 (9.3%)	446 (6.3%)	356 (11.8%)	218 (8.3%)	
High	1464 (77.4%)	4970 (83.9%)	3693 (85.8%)	5578 (88.9%)	6298 (86.1%)	6401 (90.0%)	2436 (80.7%)	2296 (87.8%)	
Overweight, n (%)	162 (8.6%)	1099 (18.6%)	604 (14.0%)	1384 (22.1%)	1171 (16.0%)	1274 (17.9%)	565 (18.7%)	431 (16.5%)	P<0.001
Obesity, n (%)	30 (1.6%)	327 (5.5%)	114 (2.6%)	423 (6.7%)	245 (3.3%)	390 (5.5%)	93 (3.1%)	128 (4.9%)	P<0.001
Mean BMI (SD)	20.8 (3.5)	22.2 (4.6)	21.8 (3.6)	23.0 (4.4)	22.3 (3.6)	22.4 (4.3)	22.7 (3.6)	22.2 (4.1)	P<0.001
Mean waist circumference, cm (SD)	81.1 (10.0)	83.3 (12.2)	82.3 (9.9)	83.1 (12.1)	83.1 (10.5)	80.9 (11.9)	84.1 (10.3)	79.7 (11.1)	P<0.001
Mean systolic blood pressure, mmHg (SD)	130.5 (21.1)	129.1 (21.0)	127.4 (16.7)	122.2 (16.7)	126.6 (14.7)	118.0 (13.4)	126.6 (13.6)	116.8 (11.9)	P<0.001
Mean diastolic blood pressure, mmHg (SD)	80.3 (11.2)	79.7 (10.5)	80.2 (9.9)	78.5 (9.6)	80.2 (9.6)	76.8 (9.1)	80.3 (8.7)	76.3 (8.4)	P<0.001
Mean fasting plasma glucose, mg/dL (SD)	92.7 (21.0)	96.6 (29.2)	92.9 (23.8)	94.1 (25.3)	91.7 (22.2)	90.4 (17.7)	91.4 (22.0)	88.7 (14.8)	P<0.001
Mean total cholesterol^b^, mg/dL (SD)	176.7 (35.7)	198.8 (43.9)	183.1 (41.5)	187.0 (42.1)	183.1 (40.0)	174.9 (37.4)	182.4 (41.4)	171.5 (39.0)	P<0.001
Mean HDL cholesterol^b^, mg/dL (SD)	42.7 (10.8)	46.6 (10.3)	42.7 (11.4)	45.6 (9.7)	41.6 (10.4)	45.6 (9.7)	39.8 (9.7)	45.4 (10.1)	P<0.001
Hypertension, n (%)	572 (30.2%)	2001 (33.8%)	1061 (24.6%)	1239 (19.7%)	1607 (22.0%)	806 (11.3%)	642 (21.3%)	228 (8.7%)	P<0.001
Diabetes, n (%)	65 (3.4%)	320 (5.4%)	149 (3.5%)	247 (3.9%)	236 (3.2%)	139 (2.0%)	111 (3.7%)	34 (1.3%)	P<0.001

**SD**: standard deviation, **HDL**: high-density lipoprotein

^a^ Presented for overall sample, not gender-specific.

^b^ Measured in subset of 3067 male participants and 4685 female participants.

**Table 3:** Distribution of cardiovascular risk factors by occupation of Solan Surveillance Study participants.

	Homemakers		Not working^a^		Low skilled		Skilled		P value^b^
	Male n = 152	Female n = 18761	Male n = 8486	Female n = 1995	Male n = 2185	Female n = 331	Male n = 5705	Female n = 842	
Current tobacco use, n (%)	35 (23.0%)	371 (2.0%)	2061 (24.3%)	10 (0.5%)	598 (27.4%)	6 (1.8%)	1138 (19.9%)	1 (0.1%)	P<0.001
Current alcohol use, n (%)	19 (12.5%)	6 (<0.1%)	1395 (16.4%)	2 (0.1%)	458 (21.0%)	1 (0.3%)	990 (17.4%)	0 (0.0%)	P<0.001
Physical activity, n (%)									P<0.001
Low	7 (4.6%)	793 (4.2%)	435 (5.1%)	123 (6.2%)	65 (3.0%)	7 (2.1%)	433 (7.6%)	25 (3.0%)	
Moderate	10 (6.6%)	1438 (7.7%)	801 (9.4%)	206 (10.3%)	112 (5.1%)	8 (2.4%)	774 (13.6%)	84 (10.0%)	
High	135 (88.8%)	16530 (88.1%)	7250 (85.4%)	1666 (83.5%)	2008 (91.9%)	316 (95.5%)	4498 (78.8%)	733 (87.1%)	
Overweight, n (%)	24 (15.8%)	3736 (19.9%)	1187 (14.0%)	181 (9.1%)	309 (14.1%)	73 (22.1%)	982 (17.2%)	198 (23.5%)	P<0.001
Obesity, n (%)	7 (4.6%)	1147 (6.1%)	238 (2.8%)	50 (2.5%)	73 (3.3%)	15 (4.5%)	164 (2.9%)	56 (6.7%)	
Mean BMI (SD)	22.2 (3.8)	22.7 (4.4)	21.7 (3.8)	20.6 (3.8)	22.2 (3.7)	22.7 (4.3)	22.6 (3.4)	23.4 (4.2)	P<0.001
Mean waist circumference, cm (SD)	81.6 (9.1)	82.7 (12.0)	82.6 (10.8)	75.4 (10.9)	82.7 (9.7)	82.5 (11.5)	83.3 (9.7)	83.5 (10.9)	P<0.001
Mean systolic blood pressure, mmHg (SD)	125.5 (15.6)	122.7 (17.6)	128.2 (17.1)	117.0 (13.4)	126.1 (14.7)	120.8 (15.5)	126.3 (14.6)	119.7 (13.7)	P<0.001
Mean diastolic blood pressure, mmHg (SD)	79.3 (9.3)	78.2 (9.8)	79.8 (10.0)	75.7 (8.5)	80.9 (9.7)	79.0 (9.1)	80.5 (9.3)	78.8 (9.0)	P<0.001
Mean fasting plasma glucose, mg/dL (SD)	94.1 (29.3)	93.5 (24.0)	92.1 (23.4)	87.6 (17.5)	91.4 (22.3)	92.4 (20.1)	92.2 (20.9)	92.5 (22.6)	P<0.001
Mean total cholesterol^c^, mg/dL (SD)	178.4 (31.2)	185.7 (41.9)	183.3 (40.5)	165.1 (38.7)	177.8 (38.7)	182.9 (40.5)	182.4 (40.6)	182.6 (39.8)	P<0.001
Mean HDL cholesterol^c^, mg/dL (SD)	42.3 (9.9)	46.0 (9.9)	42.2 (10.8)	44.3 (9.6)	41.0 (11.0)	44.0 (9.4)	41.1 (10.2)	45.0 (10.2)	P<0.001
Hypertension, n (%)	35 (23.0%)	3935 (21.0%)	2229 (26.3%)	170 (8.5%)	447 (20.5%)	56 (16.9%)	1171 (20.5%)	113 (13.4%)	P<0.001
Diabetes, n (%)	7 (4.6%)	886 (4.7%)	434 (5.1%)	33 (1.7%)	74 (3.4%)	10 (3.0%)	214 (3.8%)	32 (3.8%)	P<0.001

**SD**: standard deviation, **HDL**: high-density lipoprotein

^a^ Not working includes participants who are unemployed, students, or retired.

^b^ Presented for overall sample, not gender-specific.

^c^ Measured in subset of 3067 male participants and 4685 female participants.

The distribution of cardiovascular risk factors by monthly household income of participants is shown in Table 4. Among participants reporting monthly household income of ≤5,000 INR, tobacco and alcohol use rates were higher than in other groups. On the other hand, among households with monthly income >15,000 INR, obesity (6.7% versus 3.6% in households with monthly income ≤5,000 INR; adjusted OR 1.89, 95% CI 1.63, 2.19), hypertension (24.5% versus 19.3% in households with monthly income ≤5,000 INR; adjusted OR 1.27, 95% CI 1.13, 1.41) and diabetes (6.2% versus 3.5% in households with monthly income ≤5,000 INR; adjusted OR 1.59, 95% CI 1.31, 1.92) prevalence rates were higher compared to the lowest income group (S5 Table).

**Table 4:** Distribution of cardiovascular risk factors by household income of Solan Surveillance Study participants.

	≤5000 INR n = 11,485	5,001–10,000 INR n = 13,594	10,001–15,000 INR n = 5388	>15,000 INR n = 7990	P value
Current tobacco use, n (%)	1712 (14.9%)	1446 (10.6%)	479 (8.9%)	583 (7.3%)	P<0.001
Current alcohol use, n (%)	906 (7.9%)	1041 (7.7%)	397 (7.4%)	527 (6.6%)	P = 0.006
Physical activity, n (%)					P<0.001
Low	535 (4.7%)	835 (6.1%)	201 (3.7%)	317 (4.0%)	
Moderate	944 (8.2%)	1336 (9.8%)	475 (8.8%)	678 (8.5%)	
High	10,006 (87.1%)	11,423 (84.0%)	4712 (87.5%)	6995 (87.5%)	
Overweight, n (%)	1572 (13.7%)	2276 (16.7%)	1055 (19.6%)	1787 (22.4%)	P<0.001
Obesity, n (%)	411 (3.6%)	537 (4.0%)	269 (5.0%)	533 (6.7%)	P<0.001
Mean BMI (SD)	21.6 (4.0)	22.3 (3.9)	22.7 (4.1)	23.2 (4.2)	P<0.001
Mean waist circumference, cm (SD)	80.6 (11.1)	81.9 (11.1)	83.8 (11.3)	84.8 (11.5)	P<0.001
Mean systolic blood pressure, mmHg (SD)	123.3 (17.1)	123.8 (16.5)	125.4 (17.0)	125.7 (16.9)	P<0.001
Mean diastolic blood pressure, mmHg (SD)	78.4 (9.9)	78.9 (9.7)	79.4 (9.9)	79.7 (9.5)	P<0.001
Mean fasting plasma glucose, mg/dL (SD)	91.6 (22.4)	91.9 (21.5)	93.7 (24.9)	94.2 (25.0)	P<0.001
Mean total cholesterol^a^, mg/dL (SD)	181.4 (41.0)	181.3 (40.7)	186.0 (42.6)	187.8 (41.1)	P<0.001
Mean HDL cholesterol^a^, mg/dL (SD)	44.6 (10.5)	44.0 (10.5)	43.9 (10.0)	44.1 (10.3)	P = 0.19
Hypertension, n (%)	2222 (19.3%)	2697 (19.8%)	1277 (23.7%)	1960 (24.5%)	P<0.001
Diabetes, n (%)	403 (3.5%)	502 (3.7%)	289 (5.4%)	496 (6.2%)	P<0.001

**INR**: Indian rupee, **SD**: standard deviation, **HDL**: high-density lipoprotein

^a^ Measured in a subset of 7752 participants

The distribution of cardiovascular risk factors by household assets of participants is presented in Table 5. Both tobacco and alcohol use were highest in participants with low household assets (17.1%, and 9.4%, respectively). The proportion of participants who were overweight and obese was higher with higher levels of household assets. Furthermore, there was a graded pattern in hypertension with higher levels of hypertension in participants with highest household assets (24.9% versus 17.9% in low household assets; adjusted OR 1.42, 95% CI 1.28, 1.57, S6 Table). Diabetes prevalence followed a similar graded pattern with higher levels of diabetes in participants with highest household assets (6.9% versus 2.6% in low household assets; adjusted OR 2.32, 95% CI 1.85, 2.92, S6 Table).

**Table 5:** Distribution of cardiovascular risk factors by household assets of Solan Surveillance Study participants.

	Low n = 9639	Medium n = 10592	High n = 9018	Highest n = 9208	P value
Current tobacco use, n (%)	1651 (17.1%)	1103 (10.4%)	801 (8.9%)	665 (7.2%)	P<0.001
Current alcohol use, n (%)	905 (9.4%)	665 (6.3%)	622 (6.9%)	679 (7.4%)	P<0.001
Physical activity, n (%)					P<0.001
Low	483 (5.0%)	663 (6.3%)	339 (3.8%)	403 (4.4%)	
Moderate	876 (9.1%)	1114 (10.5%)	609 (6.8%)	834 (9.1%)	
High	8280 (85.9%)	8815 (83.2%)	8070 (89.5%)	7971 (86.6%)	
Overweight, n (%)	1161 (12.0%)	1613 (15.2%)	1768 (19.6%)	2148 (23.3%)	P<0.001
Obesity, n (%)	245 (2.5%)	385 (3.6%)	460 (5.1%)	660 (7.2%)	P<0.001
Mean BMI (SD)	21.3 (3.8)	22.0 (3.9)	22.7 (4.1)	23.4 (4.3)	P<0.001
Mean waist circumference, cm (SD)	79.7 (10.5)	81.3 (10.7)	83.5 (11.4)	85.2 (12.0)	P<0.001
Mean systolic blood pressure, mmHg (SD)	122.7 (16.8)	123.6 (16.6)	125.4 (16.6)	125.6 (17.2)	P<0.001
Mean diastolic blood pressure, mmHg (SD)	78.3 (9.9)	78.6 (9.6)	79.4 (9.6)	79.7 (9.8)	P<0.001
Mean fasting plasma glucose, mg/dL (SD)	90.6 (18.9)	91.8 (22.6)	92.7 (22.9)	95.3 (27.0)	P<0.001
Mean total cholesterol^a^, mg/dL (SD)	174.8 (39.2)	184.0 (41.8)	186.3 (41.5)	187.6 (41.1)	P<0.001
Mean HDL cholesterol^a^, mg/dL (SD)	43.9 (10.2) (n = 1748)	45.1 (10.7) (n = 2054)	44.2 (10.3) (n = 1901)	43.6 (10.3) (n = 2048)	P<0.001
Hypertension, n (%)	1726 (17.9%)	2040 (19.3%)	2093 (23.2%)	2297 (24.9%)	P<0.001
Diabetes, n (%)	255 (2.6%)	372 (3.5%)	424 (4.7%)	639 (6.9%)	P<0.001

**SD**: standard deviation, **HDL**: high-density lipoprotein

^a^ Measured in a subset of 7752 participants

**Fig 1:**
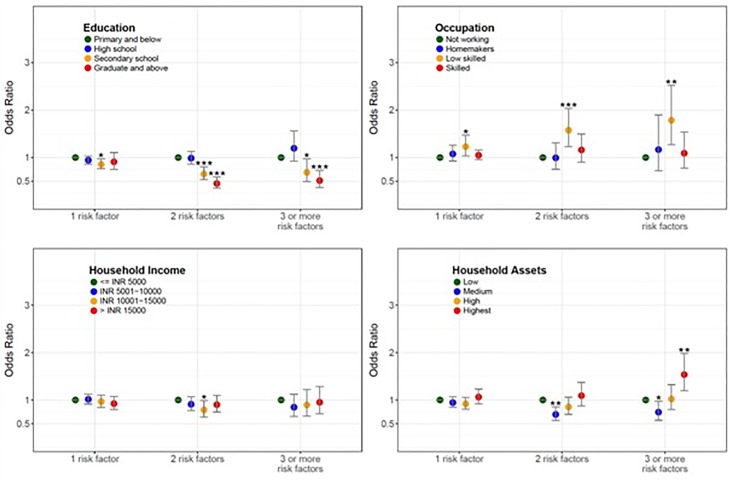
Age-, sex-, and health sub-center-adjusted association between education, occupation, household income, and household assets and number of cardiovascular disease risk factors. The age-, sex-, and health sub-center-adjusted association between socioeconomic position indicators of participant education, occupation, household income, and household assets and number of cardiovascular risk factors (tobacco use, alcohol use, low physical activity, obesity, hypertension, and diabetes). * P < 0.05, ** P < 0.01, *** P < 0.00.

The age-, sex-, and health sub-center-adjusted association between education, occupation, household income, and household assets and number of cardiovascular disease risk factors is illustrated in Fig 1. The odds of having 3 or more cardiovascular disease risk factors was highest in participants whom were low skilled workers (adjusted OR 1.79; 95% CI 1.27, 2.52) or had highest household assets (adjusted OR 1.54; 95% CI 1.19, 1.98). The age-, sex- and health sub-center-adjusted linear association between education, occupation, household income, and household assets and continuous measures of systolic blood pressure, fasting capillary glucose, BMI, total cholesterol and HDL cholesterol are illustrated in S1, S2, S3, S4 and S5 Figs **respectively**. Higher socioeconomic position measured by household assets had the largest, most consistent associations with BMI and total cholesterol followed by fasting plasma glucose and systolic blood pressure. Sensitivity analysis on the association between socioeconomic position indicators and hypertension based on measured blood pressure ≥130/80 mmHg, on blood pressure lowering medication, or self-report during the household questionnaire assessment demonstrated a consistent direction of effect as hypertension defined as measured blood pressure ≥140/90 mmHg, on blood pressure lowering medication, or self-report during the household questionnaire assessment (S1 Table).

## Discussion

In this large, representative rural population in Himachal Pradesh, India, we observed mixed patterns between the association of socioeconomic position and cardiovascular disease risk factors. Low socioeconomic position as measured by education, household income, and household assets was associated with abnormal behavioral risk factors of tobacco and alcohol use. In contrast, high socioeconomic position as measured by education, household income, and household assets was associated with abnormal clinical risk factors of obesity, hypertension, and diabetes. There was no consistent pattern amongst occupation and cardiovascular disease risk factors in rural Himachal Pradesh, which may be due to high rates of not working or homemaker status.

There are multiple, complex facets to socioeconomic position in rural India. Each socioeconomic position indictor reflects differing but related aspects of an individual’s position in society that may affect health.[11,20] For example, education as a categorical variable represents achievement of milestones (primary school, secondary school, etc.) and future earning potential but is susceptible to gender bias because women do not have equal access to education compared with men in rural India.[21] Further, occupation reflects social standing in society; however, homemakers, students, retired, and unemployed are often inadequately categorized. Self-reported monthly household income may be inconsistent as income can vary month to month in rural agrarian communities. Household assets reflect current material wealth and may not capture generational wealth. Other aspects of socioeconomic position including life course and neighborhood socioeconomic status are important, although were not measured in this study. Evaluating multiple socioeconomic position indicators provides a more comprehensive representation of the social determinants of health in rural India, and national policies to reduce health disparities must simultaneously address multiple indicators.[22,23] Our findings are consistent with prior literature on the high prevalence of modifiable, unhealthy behaviors such as tobacco and alcohol use among individuals with lower socioeconomic position. A 2017 systematic review including 75 studies representing 2,135,314 individuals from 39 low-income and lower-middle-income countries demonstrated lower socioeconomic groups had a significantly higher prevalence of tobacco and alcohol use than higher socioeconomic groups.[24] Education was the strongest predictor of tobacco use compared to other socioeconomic position indicators; individuals with no formal education were 1.8 to 6.5 times more likely to smoke than individuals with at least a secondary education, which is congruent with our findings.[24] In a 2005 cross-sectional study of 4,535 adults in rural Andhra Pradesh, individuals with no education were more likely to be current smokers (57.7% vs 39.5%, P <0.001) and use alcohol (36.8% vs 25.5%, P <0.001) compared to those with some education.[12] Similarly, the prevalence of tobacco and alcohol use was more common in low socioeconomic position individuals in a cross-sectional surveillance report conducted from 2005 to 2007 of 1,983 individuals from rural villages in 18 states in India.[25] This social patterning of higher prevalence of tobacco use amongst the lower socioeconomic strata is consistent with other studies conducted in India and similar to this study’s findings from rural Himachal Pradesh.[26–30] Tobacco use is one of the strongest modifiable risk factors for cardiovascular disease and causes the largest number of premature deaths in India.[29–31] This social patterning of higher tobacco use amongst individuals of lower socioeconomic position may be related to lower locus of control leading to higher initiation and consumption rates, and lower cessation rates due, at least in part, to lower affordability for tobacco cessation treatment.[32,33] A deeper understanding of this social patterning can help develop targeted tobacco control efforts in India’s resource-constrained health system.

Our findings are also consistent with prior literature showing the high prevalence of clinical risk factors of obesity, hypertension, and diabetes among individuals with higher socioeconomic position in south Asia.[10,12,25,34,35] A 2015–2016 nationally representative sample from the National Family Health Survey (n = 757,958) demonstrated an 8.8-fold higher odds of obesity among individuals in the highest quintile of income compared with individuals in the lowest quintile, though the odds were lower when comparing other markers of socioeconomic position such as education or caste.[19] In a 2012 to 2014 nationally representative study of 1.3 million adults in India, being in the richest household wealth quintile compared with being in the poorest quintile was associated with higher probability of hypertension (4.2%, 95% CI 3.7%, 4.6%) and diabetes (2.8%, 95% CI 2.5%, 3.1%) amongst individuals living in rural areas.[35] A 2012 analysis using nationally representative cross-sectional data with 168,135 individuals in India demonstrated that those in the richest household wealth quintile had 2.6 (95% credible interval: 2.0, 3.4) times higher odds of having diabetes than the poorest household wealth quintile.[36] In a 2010 cross-sectional surveillance study of 1,983 individuals from rural villages in India, higher rates of overweight (men 25.4%, women 35.0%), hypertension (men 20.8%, women 25.3%) and diabetes (men 8.0%) were noted amongst rural participants with higher socioeconomic position, which parallels our findings of participants with highest household assets more likely to be overweight (23.3%), have hypertension (24.9%) and diabetes (6.9%).[25] The epidemiological transition of higher clinical cardiovascular disease risk factors amongst those with higher wealth has been studied in urban India, and our analysis suggests this transition may also be occurring in rural India where the majority of the country’s population resides.[10]

We present the linear association between socioeconomic position indicators and measured blood pressure, fasting plasma glucose, measured BMI, total cholesterol and HDL cholesterol, which is lacking in prior literature on cardiovascular disease risk factors in rural India.[12,25] The current study showed a consistent step-wise increase in systolic blood pressure, fasting plasma glucose, BMI, and total cholesterol with higher wealth as measured by household income and household assets in rural Himachal Pradesh. Broad-based policies that support cardiovascular health promotion and primordial prevention, including best buys for preventing noncommunicable diseases outlined by the World Health Organization, may help prevent not only disease incidence but also risk factor development. These data might also help tailor interventions such as tobacco and alcohol cessation, dietary modifications or task-shifting for risk factor management with clinical decision support systems to target the highest risk groups.[37]

Our study has several important strengths. We present data from a large, representative sampling frame of rural Himachal Pradesh. We used multiple indicators to characterize socioeconomic position and the association with cardiovascular disease risk factors compared to prior research.[12,25,26,36] Furthermore, there were few missing data (3.9%) in the exposure and outcome data. We used objective measurements of anthropometry, blood pressure, fasting plasma glucose, and fasting lipid panel to define the cardiovascular disease risk factors, which have been demonstrated to be more accurate than self-report alone.[38]

Our study also has important limitations. First, the exposures of participant education, participant occupation, household income, and household assets were assessed through self-report and may be susceptible to reporting bias. However, triangulation of these self-reported data with objective measures of socioeconomic position on a sample this large would be infeasible, which supports the use of multiple indicators of socioeconomic position. Second, there may be unmeasured confounders such as unmeasured socioeconomic circumstances or behaviors that were not incorporated into our regression models and may influence the results. Third, we present data from one state in India, which may not be generalizable to the entire country but does offer novel insights. Fourth, the cross-sectional study design limits causal inference for the proposed relationships; however, previous research suggests that socioeconomic position has an independent, causal relationship with cardiovascular disease risk factors and cardiovascular disease driven by lifecourse exposure to deprivation leading to changes in behaviors, disease susceptibility, prevention, and treatment, and access to health care.[39]

## Conclusion

In this large, representative rural population in Himachal Pradesh, India, we observed mixed patterns between the association of socioeconomic position and cardiovascular disease risk factors. Individuals with lower socioeconomic position were more likely to have abnormal behavioral risk factors, and individuals with higher socioeconomic position were more likely to have abnormal clinical risk factors. Thus, context is essential in understanding the relationship between disadvantage and disease. We demonstrate that the patterns of higher prevalence of obesity, hypertension, and diabetes amongst the wealthier strata observed in urban India are also observed in rural India.[10] A better understanding of the social patterning of disease can guide cardiovascular disease prevention efforts to target higher risk groups in rural India.

## Supporting information

S1 Table Association between socioeconomic position indicators and hypertension based on measured blood pressure ≥130/80 mmHg. (DOCX)

S2 Table Characteristics of Solan Surveillance Study participants with missing data. (DOCX)

S3 Table Association between education and cardiovascular disease risk factors. (DOCX)

S4 Table Association between occupation and cardiovascular disease risk factors. (DOCX)

S5 Table Association between household income and cardiovascular disease risk factors. (DOCX)

S6 Table Association between household assets and cardiovascular disease risk factors. (DOCX)

S1 Fig Age-, sex- and health sub-center-adjusted association between socioeconomic position indicators and systolic blood pressure. (DOCX)

S2 Fig Age-, sex- and health sub-center-adjusted association between socioeconomic position indictors and fasting capillary glucose. (DOCX)

S3 Fig Age-, sex- and health sub-center-adjusted association between socioeconomic position indicators and body mass index. (DOCX)

S4 Fig Age-, sex- and health sub-center-adjusted association between socioeconomic position indicators and total cholesterol. (DOCX)

S5 Fig Age-, sex- and health sub-center-adjusted association between socioeconomic position indicators and HDL cholesterol. (DOCX)
