# Supplementary material for: Association between socioeconomic position and cardiovascular disease risk factors in rural north India: The Solan Surveillance Study
Source: PLoS One. 2019 Jul 8;14(7):e0217834. doi: 10.1371/journal.pone.0217834 (PMC6613705; doi:10.1371/journal.pone.0217834)
Supplement: S1 Table — (DOCX) [file pone.0217834.s001.docx]

**S1 Table.** Association between socioeconomic position indicators and hypertension based on measured blood pressure >130/80 mmHg.

| **Socioeconomic position** | | **Model 1**^a^ | | **Model 2**^b^ | | **Model 3**^c^ | |
| --- | --- | --- | --- | --- | --- | --- | --- |
|  |  | **OR** | **95% CI** | **OR** | **95% CI** | **OR** | **95% CI** |
| Education | Primary and below | 1.00 | - | 1.00 | - | 1.00 | - |
|  | High school | 0.70 | 0.66, 0.75 | 1.07 | 1.00, 1.14 | 1.07 | 0.99, 1.16 |
|  | Secondary school | 0.59 | 0.55, 0.62 | 1.16 | 1.08, 1.24 | 1.16 | 1.03, 1.29 |
|  | Graduate and above | 0.60 | 0.56, 0.64 | 1.31 | 1.21, 1.43 | 1.31 | 1.16, 1.49 |
| Occupation | Not working | 1.00 | - | 1.00 | - | 1.00 | - |
|  | Homemakers | 0.81 | 0.77, 0.85 | 1.04 | 0.96, 1.13 | 1.04 | 0.94, 1.15 |
|  | Low skilled | 1.17 | 1.07, 1.28 | 1.32 | 1.20, 1.44 | 1.32 | 1.15, 1.51 |
|  | Skilled | 1.05 | 0.98, 1.11 | 1.06 | 1.00, 1.14 | 1.06 | 0.88, 1.29 |
| Household income | ≤5,000 INR | 1.00 | - | 1.00 | - | 1.00 | - |
|  | 5,001-10,000 INR | 1.00 | 0.94, 1.06 | 1.03 | 0.97, 1.09 | 1.03 | 0.89, 1.19 |
|  | 10,001-15,000 INR | 1.20 | 1.11, 1.29 | 1.18 | 1.09, 1.28 | 1.18 | 1.01, 1.37 |
|  | >15,000 INR | 1.27 | 1.19, 1.36 | 1.24 | 1.15, 1.33 | 1.24 | 1.08, 1.42 |
| Household assets | Low | 1.00 | - | 1.00 | - | 1.00 | - |
|  | Medium | 1.10 | 1.04, 1.16 | 1.05 | 0.99, 1.12 | 1.05 | 0.93, 1.20 |
|  | High | 1.35 | 1.28, 1.43 | 1.29 | 1.22, 1.38 | 1.29 | 1.16, 1.45 |
|  | Highest | 1.41 | 1.33, 1.49 | 1.34 | 1.26, 1.42 | 1.34 | 1.22, 1.47 |
| **OR:** odds ratio; **CI:** confidence interval; **INR:** Indian rupees  ^a^Unadjusted model; ^b^Adjusted for age and sex; ^c^Adjusted for age, sex, and health sub-center clustering | | | | | | | |
