# Supplementary material for: Association between socioeconomic position and cardiovascular disease risk factors in rural north India: The Solan Surveillance Study
Source: PLoS One. 2019 Jul 8;14(7):e0217834. doi: 10.1371/journal.pone.0217834 (PMC6613705; doi:10.1371/journal.pone.0217834)
Supplement: S2 Table — (DOCX) [file pone.0217834.s002.docx]

**S2 Table**. Characteristics of Solan Surveillance Study participants with missing data.

| **Characteristic** | **Complete Case Analysis** | **Missing** | **P value** |
| --- | --- | --- | --- |
| No. of participants | 38,457 | 1560 |  |
| Age group, n (%) |  |  |  |
| 20-29 years | 9327 (24.3%) | 376 (24.1%) | 0.01 |
| 30-39 years | 9367 (24.4%) | 359 (23.0%) |  |
| 40-49 years | 7574 (19.7%) | 315 (20.2%) |  |
| 50-59 years | 5549 (14.4%) | 224 (14.4%) |  |
| 60-69 years | 3827 (10.0%) | 135 (8.7%) |  |
| ≥70 years | 2813 (7.3%) | 151 (9.7%) |  |
| Mean age, years (SD) | 42.7 (15.9) | 43.4 (17.0) | 0.06 |
| Education^a^, n (%) |  |  |  |
| Primary school and below | 7815 (20.3%) | 386 (24.7%) | <0.001 |
| High school | 10,581 (27.5%) | 465 (29.8%) |  |
| Secondary school | 14,428 (37.5%) | 517 (33.1%) |  |
| Graduate & above | 5633 (14.6%) | 192 (12.3%) |  |
| Mean years of formal education (SD) | 8.3 (5.0) | 7.7 (5.0) | <0.001 |
| Occupation^b^, n (%) |  |  |  |
| Homemaker | 18,913 (49.2%) | 745 (47.8%) | <0.001 |
| Not working | 10,481 (27.3%) | 376 (24.1%) |  |
| Low skilled | 2516 (6.5%) | 148 (9.5%) |  |
| Skilled | 6547 (17.0%) | 275 (17.6%) |  |
| Monthly household income, n (%) |  |  |  |
| ≤INR 5,000 | 11,485 (29.9%) | 543 (34.8%) | <0.001 |
| INR 5,001 – 10,000 | 13,594 (35.3%) | 559 (35.8%) |  |
| INR 10,001 – 15,000 | 5388 (14.0%) | 183 (11.7%) |  |
| >INR 15,000 | 7990 (20.8%) | 270 (17.3%) |  |
| Mean monthly household income, INR (SD) | 12,355.9 (15404.0) | 11203.0 (12100.2) | 0.004 |
| Household asset quartile, n (%) |  |  |  |
| Low | 9510 (24.7%) | 527 (33.8%) | <0.001 |
| Medium | 10,720 (27.9%) | 381 (24.4%) |  |
| High | 9019 (23.5%) | 258 (16.5%) |  |
| Highest | 9208 (23.9%) | 322 (20.6%) |  |
| Current tobacco use, n (%) | 4220 (11.0%) | 191 (12.2%) | 0.12 |
| Current alcohol use, n (%) | 2871 (7.5%) | 129 (8.3%) | 0.24 |
| **SD:** standard deviation, **INR**: Indian rupee  ^a^ Primary school and below: up to class IV, literate with no formal education, or illiterate; High school: class V to IX; Secondary school: class X to XII; Graduate & above: bachelor of arts, bachelor of science, bachelor of commerce, diploma, or professional degree.  ^b^ Homemaker: a person who manages the home; Not working: unemployed, retired, or student; Low skilled: manual laborer, rickshaw driver, carpenter, etc.; Skilled: farmer, business owner, teacher, etc. | | | |
