# Supplementary material for: Association between socioeconomic position and cardiovascular disease risk factors in rural north India: The Solan Surveillance Study
Source: PLoS One. 2019 Jul 8;14(7):e0217834. doi: 10.1371/journal.pone.0217834 (PMC6613705; doi:10.1371/journal.pone.0217834)
Supplement: S3 Table — (DOCX) [file pone.0217834.s003.docx]

**S3 Table.** Association between education and cardiovascular disease risk factors.

| **CVD Risk Factors** | | **Model 1**^a^ | | **Model 2**^b^ | | **Model 3**^c^ | |
| --- | --- | --- | --- | --- | --- | --- | --- |
|  |  | **OR** | **95% CI** | **OR** | **95% CI** | **OR** | **95% CI** |
| Current tobacco use | Primary and below | 1.00 | - | 1.00 | - | 1.00 | - |
|  | High school | 0.89 | 0.82, 0.97 | 0.51 | 0.46, 0.57 | 0.51 | 0.45, 0.58 |
|  | Secondary school | 0.49 | 0.45, 0.54 | 0.22 | 0.20, 0.25 | 0.22 | 0.19, 0.26 |
|  | Graduate and above | 0.25 | 0.22, 0.29 | 0.11 | 0.09, 0.12 | 0.11 | 0.09, 0.13 |
| Current alcohol use | Primary and below | 1.00 | - | 1.00 | - | 1.00 | - |
|  | High school | 1.52 | 1.36, 1.70 | 1.01 | 0.89, 1.15 | 1.01 | 0.83, 1.24 |
|  | Secondary school | 1.10 | 0.98, 1.22 | 0.60 | 0.52, 0.68 | 0.60 | 0.46, 0.77 |
|  | Graduate and above | 0.80 | 0.69, 0.93 | 0.42 | 0.35, 0.50 | 0.42 | 0.32, 0.55 |
| Low physical activity | Primary and below | 1.00 | - | 1.00 | - | 1.00 | - |
|  | High school | 0.65 | 0.57, 0.74 | 0.74 | 0.64, 0.85 | 0.74 | 0.60, 0.91 |
|  | Secondary school | 0.64 | 0.56, 0.72 | 0.79 | 0.68, 0.92 | 0.79 | 0.61, 1.04 |
|  | Graduate and above | 0.89 | 0.77, 1.03 | 1.15 | 0.97, 1.37 | 1.15 | 0.79, 1.68 |
| Obesity | Primary and below | 1.00 | - | 1.00 | - | 1.00 | - |
|  | High school | 1.12 | 0.97, 1.28 | 1.94 | 1.67, 2.26 | 1.94 | 1.69, 2.24 |
|  | Secondary school | 0.96 | 0.84, 1.10 | 2.34 | 1.99, 2.75 | 2.34 | 1.84, 2.98 |
|  | Graduate and above | 0.85 | 0.72, 1.01 | 2.33 | 1.91, 2.85 | 2.33 | 1.85, 2.94 |
| Hypertension | Primary and below | 1.00 | - | 1.00 | - | 1.00 | - |
|  | High school | 0.57 | 0.53, 0.60 | 1.17 | 1.08, 1.26 | 1.17 | 1.07, 1.27 |
|  | Secondary school | 0.41 | 0.38, 0.44 | 1.29 | 1.19, 1.40 | 1.29 | 1.14, 1.46 |
|  | Graduate and above | 0.37 | 0.34, 0.41 | 1.39 | 1.25, 1.54 | 1.39 | 1.19, 1.62 |
| Diabetes | Primary and below | 1.00 | - | 1.00 | - | 1.00 | - |
|  | High school | 0.75 | 0.65, 0.87 | 1.71 | 1.45, 2.00 | 1.71 | 1.39, 2.09 |
|  | Secondary school | 0.52 | 0.45, 0.60 | 1.85 | 1.55, 2.21 | 1.85 | 1.29, 2.65 |
|  | Graduate and above | 0.51 | 0.42, 0.62 | 2.15 | 1.71, 2.69 | 2.15 | 1.59, 2.90 |
| **CVD:** cardiovascular disease; **OR:** odds ratio; **CI:** confidence interval  ^a^Unadjusted model; ^b^Adjusted for age and sex; ^c^Adjusted for age, sex, and health sub-center clustering | | | | | | | |
