# Supplementary material for: Association between socioeconomic position and cardiovascular disease risk factors in rural north India: The Solan Surveillance Study
Source: PLoS One. 2019 Jul 8;14(7):e0217834. doi: 10.1371/journal.pone.0217834 (PMC6613705; doi:10.1371/journal.pone.0217834)
Supplement: S4 Table — (DOCX) [file pone.0217834.s004.docx]

**S4 Table.** Association between occupation and cardiovascular disease risk factors.

| **CVD Risk Factors** | | **Model 1**^a^ | | **Model 2**^b^ | | **Model 3**^c^ | |
| --- | --- | --- | --- | --- | --- | --- | --- |
|  |  | **OR** | **95% CI** | **OR** | **95% CI** | **OR** | **95% CI** |
| Current tobacco use | Not working | 1.00 | - | 1.00 | - | 1.00 | - |
|  | Homemakers | 0.09 | 0.08, 0.10 | 1.48 | 1.14, 1.93 | 1.48 | 1.12, 1.96 |
|  | Low skilled | 1.28 | 1.16, 1.42 | 1.60 | 1.44, 1.79 | 1.60 | 1.20, 2.15 |
|  | Skilled | 0.86 | 0.79, 0.93 | 0.93 | 0.85, 1.01 | 0.93 | 0.74, 1.16 |
| Current alcohol use | Not working | 1.00 | - | 1.00 | - | 1.00 | - |
|  | Homemakers | 0.01 | 0.01, 0.01 | 0.68 | 0.42, 1.10 | 0.68 | 0.38, 1.20 |
|  | Low skilled | 1.45 | 1.29, 1.63 | 1.70 | 1.50, 1.92 | 1.70 | 1.29, 2.23 |
|  | Skilled | 1.16 | 1.06, 1.27 | 1.23 | 1.12, 1.35 | 1.23 | 0.94, 1.61 |
| Low physical activity | Not working | 1.00 | - | 1.00 | - | 1.00 | - |
|  | Homemakers | 0.79 | 0.70, 0.88 | 0.75 | 0.63, 0.91 | 0.75 | 0.59, 0.97 |
|  | Low skilled | 0.52 | 0.41, 0.67 | 0.58 | 0.45, 0.75 | 0.58 | 0.38, 0.88 |
|  | Skilled | 1.34 | 1.18, 1.52 | 1.41 | 1.24, 1.61 | 1.41 | 0.97, 2.06 |
| Obesity | Not working | 1.00 | - | 1.00 | - | 1.00 | - |
|  | Homemakers | 2.30 | 2.02, 2.62 | 1.53 | 1.24, 1.87 | 1.53 | 1.32, 1.77 |
|  | Low skilled | 1.28 | 1.01, 1.64 | 1.46 | 1.15, 1.87 | 1.46 | 1.13, 1.89 |
|  | Skilled | 1.23 | 1.03, 1.47 | 1.34 | 1.12, 1.60 | 1.34 | 1.09, 1.64 |
| Hypertension | Not working | 1.00 | - | 1.00 | - | 1.00 | - |
|  | Homemakers | 0.90 | 0.90, 0.95 | 1.21 | 1.07, 1.37 | 1.21 | 1.04, 1.42 |
|  | Low skilled | 0.84 | 0.76, 0.94 | 1.28 | 1.14, 1.43 | 1.28 | 1.11, 1.47 |
|  | Skilled | 0.82 | 0.76, 0.89 | 1.04 | 0.96, 1.13 | 1.04 | 0.84, 1.30 |
| Diabetes | Not working | 1.00 | - | 1.00 | - | 1.00 | - |
|  | Homemakers | 1.09 | 0.95, 1.24 | 1.19 | 0.91, 1.55 | 1.19 | 0.91, 1.55 |
|  | Low skilled | 0.82 | 0.63, 1.06 | 1.33 | 1.02, 1.75 | 1.33 | 0.98, 1.82 |
|  | Skilled | 0.90 | 0.75, 1.07 | 1.24 | 1.04, 1.50 | 1.24 | 0.96, 1.62 |
| **CVD:** cardiovascular disease; **OR:** odds ratio; **CI:** confidence interval  ^a^Unadjusted model; ^b^Adjusted for age and sex; ^c^Adjusted for age, sex, and health sub-center clustering | | | | | | | |
