# Supplementary material for: Association between socioeconomic position and cardiovascular disease risk factors in rural north India: The Solan Surveillance Study
Source: PLoS One. 2019 Jul 8;14(7):e0217834. doi: 10.1371/journal.pone.0217834 (PMC6613705; doi:10.1371/journal.pone.0217834)
Supplement: S5 Table — (DOCX) [file pone.0217834.s005.docx]

**S5 Table.** Association between household income and cardiovascular disease risk factors.

| **CVD Risk Factors** | | **Model 1**^a^ | | **Model 2**^b^ | | **Model 3**^c^ | |
| --- | --- | --- | --- | --- | --- | --- | --- |
|  |  | **OR** | **95% CI** | **OR** | **95% CI** | **OR** | **95% CI** |
| Current tobacco use | ≤5,000 INR | 1.00 | - | 1.00 | - | 1.00 | - |
|  | 5,001-10,000 INR | 0.68 | 0.63, 0.73 | 0.60 | 0.55, 0.65 | 0.60 | 0.52, 0.68 |
|  | 10,001-15,000 INR | 0.56 | 0.50, 0.62 | 0.45 | 0.40, 0.50 | 0.45 | 0.38, 0.52 |
|  | >15,000 INR | 0.45 | 0.41, 0.50 | 0.35 | 0.32, 0.39 | 0.35 | 0.29, 0.43 |
| Current alcohol use | ≤5,000 INR | 1.00 | - | 1.00 | - | 1.00 | - |
|  | 5,001-10,000 INR | 0.97 | 0.88, 1.06 | 0.92 | 0.83, 1.01 | 0.92 | 0.79, 1.06 |
|  | 10,001-15,000 INR | 0.93 | 0.82, 1.05 | 0.85 | 0.74, 0.97 | 0.85 | 0.73, 0.99 |
|  | >15,000 INR | 0.83 | 0.74, 0.92 | 0.75 | 0.66, 0.84 | 0.75 | 0.64, 0.88 |
| Low physical activity | ≤5,000 INR | 1.00 | - | 1.00 | - | 1.00 | - |
|  | 5,001-10,000 INR | 1.34 | 1.20, 1.50 | 1.34 | 1.20, 1.50 | 1.34 | 0.88, 2.06 |
|  | 10,001-15,000 INR | 0.79 | 0.67, 0.94 | 0.77 | 0.65, 0.91 | 0.77 | 0.62, 0.97 |
|  | >15,000 INR | 0.85 | 0.73, 0.98 | 0.82 | 0.71, 0.94 | 0.82 | 0.62, 1.09 |
| Obesity | ≤5,000 INR | 1.00 | - | 1.00 | - | 1.00 | - |
|  | 5,001-10,000 INR | 1.11 | 0.97, 1.26 | 1.13 | 0.99, 1.29 | 1.13 | 0.99, 1.30 |
|  | 10,001-15,000 INR | 1.42 | 1.21, 1.66 | 1.40 | 1.19, 1.64 | 1.40 | 1.13, 1.73 |
|  | >15,000 INR | 1.93 | 1.69, 2.20 | 1.89 | 1.65, 2.16 | 1.89 | 1.63, 2.19 |
| Hypertension | ≤5,000 INR | 1.00 | - | 1.00 | - | 1.00 | - |
|  | 5,001-10,000 INR | 1.03 | 0.97, 1.10 | 1.06 | 0.99, 1.13 | 1.06 | 0.93, 1.21 |
|  | 10,001-15,000 INR | 1.30 | 1.20, 1.40 | 1.22 | 1.12, 1.33 | 1.22 | 1.08, 1.39 |
|  | >15,000 INR | 1.36 | 1.27, 1.45 | 1.27 | 1.17, 1.36 | 1.27 | 1.13, 1.41 |
| Diabetes | ≤5,000 INR | 1.00 | - | 1.00 | - | 1.00 | - |
|  | 5,001-10,000 INR | 1.01 | 0.87, 1.18 | 1.04 | 0.89, 1.21 | 1.04 | 0.88, 1.22 |
|  | 10,001-15,000 INR | 1.47 | 1.23, 1.75 | 1.37 | 1.15, 1.64 | 1.37 | 1.12, 1.67 |
|  | >15,000 INR | 1.72 | 1.48, 2.00 | 1.59 | 1.36, 1.85 | 1.59 | 1.31, 1.92 |
| **CVD:** cardiovascular disease; **OR:** odds ratio; **CI:** confidence interval; **INR**: Indian rupees  ^a^Unadjusted model; ^b^Adjusted for age and sex; ^c^Adjusted for age, sex, and health sub-center clustering | | | | | | | |
