# Supplementary material for: Association between socioeconomic position and cardiovascular disease risk factors in rural north India: The Solan Surveillance Study
Source: PLoS One. 2019 Jul 8;14(7):e0217834. doi: 10.1371/journal.pone.0217834 (PMC6613705; doi:10.1371/journal.pone.0217834)
Supplement: S6 Table — (DOCX) [file pone.0217834.s006.docx]

**S6 Table.** Association between household assets and cardiovascular disease risk factors.

| **CVD Risk Factors** | | **Model 1**^a^ | | **Model 2**^b^ | | **Model 3**^c^ | |
| --- | --- | --- | --- | --- | --- | --- | --- |
|  |  | **OR** | **95% CI** | **OR** | **95% CI** | **OR** | **95% CI** |
| Current tobacco use | Low | 1.00 | - | 1.00 | - | 1.00 | - |
|  | Medium | 0.56 | 0.52, 0.61 | 0.48 | 0.44, 0.52 | 0.48 | 0.41, 0.55 |
|  | High | 0.47 | 0.43, 0.52 | 0.37 | 0.33, 0.41 | 0.37 | 0.31, 0.44 |
|  | Highest | 0.38 | 0.34, 0.41 | 0.28 | 0.26, 0.32 | 0.28 | 0.24, 0.34 |
| Current alcohol use | Low | 1.00 | - | 1.00 | - | 1.00 | - |
|  | Medium | 0.65 | 0.58, 0.72 | 0.60 | 0.54, 0.67 | 0.60 | 0.51, 0.71 |
|  | High | 0.72 | 0.64, 0.80 | 0.65 | 0.58, 0.73 | 0.65 | 0.54, 0.77 |
|  | Highest | 0.77 | 0.69, 0.85 | 0.70 | 0.62, 0.78 | 0.70 | 0.59, 0.82 |
| Low physical activity | Low | 1.00 | - | 1.00 | - | 1.00 | - |
|  | Medium | 1.27 | 1.12, 1.43 | 1.24 | 1.10, 1.40 | 1.24 | 0.96, 1.61 |
|  | High | 0.74 | 0.64, 0.85 | 0.72 | 0.62, 0.83 | 0.72 | 0.48, 1.08 |
|  | Highest | 0.87 | 0.76, 0.99 | 0.84 | 0.73, 0.96 | 0.84 | 0.57, 1.22 |
| Obesity | Low | 1.00 | - | 1.00 | - | 1.00 | - |
|  | Medium | 1.45 | 1.23, 1.70 | 1.41 | 1.19, 1.65 | 1.41 | 1.20, 1.64 |
|  | High | 2.06 | 1.76, 2.41 | 2.00 | 1.70, 2.34 | 2.00 | 1.64, 2.42 |
|  | Highest | 2.96 | 2.55, 3.44 | 2.87 | 2.47, 3.34 | 2.87 | 2.39, 3.45 |
| Hypertension | Low | 1.00 | - | 1.00 | - | 1.00 | - |
|  | Medium | 1.09 | 1.02, 1.17 | 1.02 | 0.95, 1.10 | 1.02 | 0.88, 1.18 |
|  | High | 1.39 | 1.29, 1.49 | 1.29 | 1.19, 1.39 | 1.29 | 1.17, 1.41 |
|  | Highest | 1.52 | 1.42, 1.64 | 1.42 | 1.31, 1.53 | 1.42 | 1.28, 1.57 |
| Diabetes | Low | 1.00 | - | 1.00 | - | 1.00 | - |
|  | Medium | 1.28 | 1.07, 1.53 | 1.19 | 1.00, 1.43 | 1.19 | 0.98, 1.46 |
|  | High | 1.62 | 1.35, 1.93 | 1.47 | 1.23, 1.77 | 1.47 | 1.17, 1.86 |
|  | Highest | 2.53 | 2.15, 2.98 | 2.32 | 1.97, 2.75 | 2.32 | 1.85, 2.92 |
| **CVD:** cardiovascular disease; **OR:** odds ratio; **CI:** confidence interval  ^a^Unadjusted model; ^b^Adjusted for age and sex; ^c^Adjusted for age, sex, and health sub-center clustering | | | | | | | |
