# Supplementary material for: Association between socioeconomic position and cardiovascular disease risk factors in rural north India: The Solan Surveillance Study
Source: PLoS One. 2019 Jul 8;14(7):e0217834. doi: 10.1371/journal.pone.0217834 (PMC6613705; doi:10.1371/journal.pone.0217834)
Supplement: S1 Fig — (DOCX) [file pone.0217834.s007.docx]

**S1 Figure.** Age-, sex- and health sub-center-adjusted association between socioeconomic position indicators and systolic blood pressure.

**
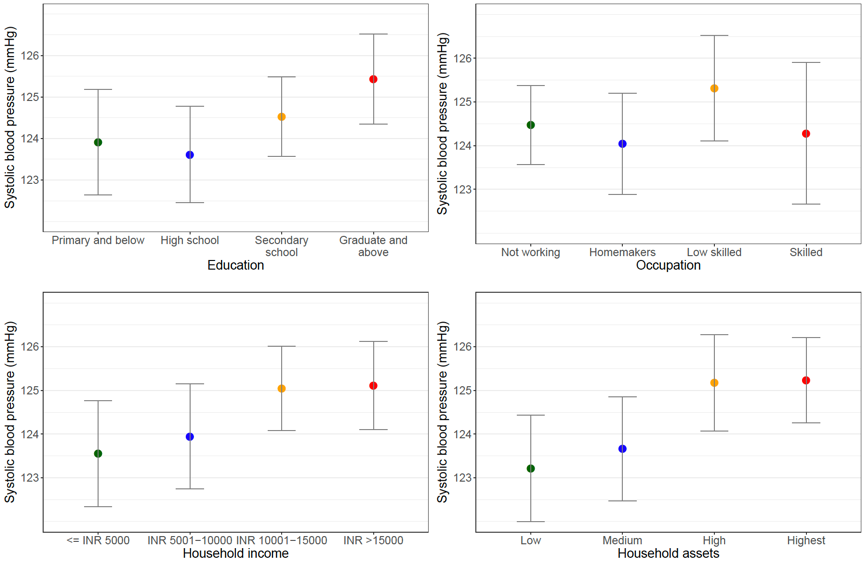
**

The linear association between socioeconomic position indicators of participant education, participant occupation, household monthly income, and household assets and systolic blood pressure (mmHg) in 38,457 participants in the Solan Surveillance Study.
