# Supplementary material for: Association between socioeconomic position and cardiovascular disease risk factors in rural north India: The Solan Surveillance Study
Source: PLoS One. 2019 Jul 8;14(7):e0217834. doi: 10.1371/journal.pone.0217834 (PMC6613705; doi:10.1371/journal.pone.0217834)
Supplement: S5 Fig — (DOCX) [file pone.0217834.s011.docx]

**S5 Figure.** Age-, sex- and health sub-center-adjusted association between socioeconomic position indicators and HDL cholesterol.


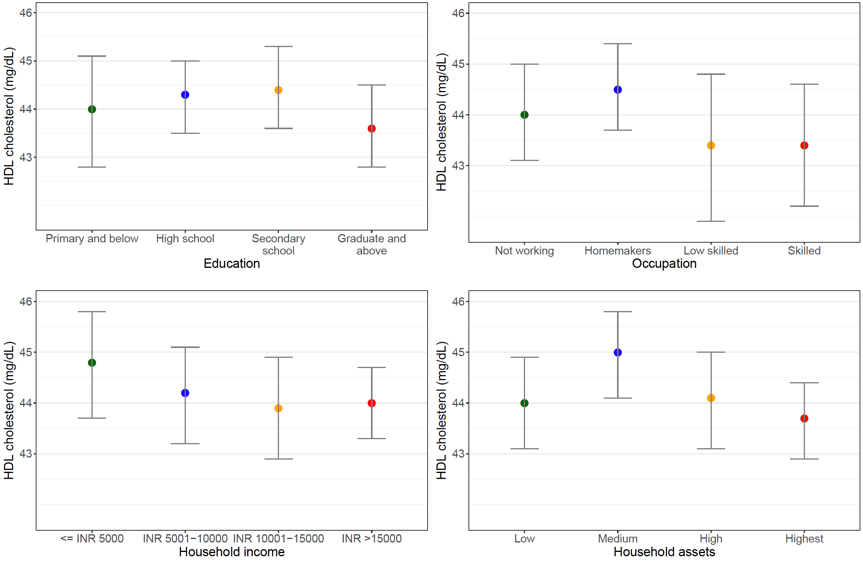


The linear association between socioeconomic position indicators of participant education, participant occupation, household monthly income, and household assets and high-density lipoprotein (HDL) cholesterol (mg/dL) in 7,751 participants in the Solan Surveillance Study.
